# Management of Advanced Prostate Cancer in the Precision Oncology Era

**DOI:** 10.3390/cancers15092552

**Published:** 2023-04-29

**Authors:** Claire M. Gillette, Gabriel A. Yette, Scott D. Cramer, Laura S. Graham

**Affiliations:** 1 Department of Pharmacology, University of Colorado Anschutz Medical Campus, Aurora, CO 80045, USA; claire.gillette@cuanschutz.edu (C.M.G.);; 2 Division of Medical Oncology, University of Colorado Anschutz Medical Campus, Aurora, CO 80045, USA

**Keywords:** metastatic prostate cancer, advanced prostate cancer, biomarkers, genomic selection, chemotherapy, PARP inhibitors, immunotherapy, PSMA, theranostics, radioligand therapy

## Abstract

**Simple Summary:**

This review article details new precision oncology-based therapeutics used to treat advanced prostate cancer. Metastatic castration-resistant prostate cancer remains an incurable diagnosis, however newly identified biomarkers have expanded treatment repertories for some patients with progressive disease. Advancements in immune checkpoint inhibitors, therapeutics that exploit DNA damage response deficiencies, and new innovations in radioligand therapies and theranostics are detailed. This article highlights the potential and real-world application of precision oncology to improve outcomes for patients with prostate cancer by tailoring treatment to the individual characteristics of each patient’s tumor.

**Abstract:**

Prostate cancer (PC) is the second leading cause of cancer death in men in the United States. While diversified and improved treatment options for aggressive PC have improved patient outcomes, metastatic castration-resistant prostate cancer (mCRPC) remains incurable and an area of investigative therapeutic interest. This review will cover the seminal clinical data supporting the indication of new precision oncology-based therapeutics and explore their limitations, present utility, and potential in the treatment of PC. Systemic therapies for high-risk and advanced PC have experienced significant development over the past ten years. Biomarker-driven therapies have brought the field closer to the goal of being able to implement precision oncology therapy for every patient. The tumor agnostic approval of pembrolizumab (a PD-1 inhibitor) marked an important advancement in this direction. There are also several PARP inhibitors indicated for patients with DNA damage repair deficiencies. Additionally, theranostic agents for both imaging and treatment have further revolutionized the treatment landscape for PC and represent another advancement in precision medicine. Radiolabeled prostate-specific membrane antigen (PSMA) PET/CT is rapidly becoming a standard of care for diagnosis, and PSMA-targeted radioligand therapies have gained recent FDA approval for metastatic prostate cancer. These advances in precision-based oncology are detailed in this review.

## 1. Introduction

Prostate cancer (PC) is the second leading cause of cancer death in men in the United States [1]. In 2022, it was estimated that PC comprised 14% of all new cancer cases in the United States and accounted for 5.7% of all cancer deaths. PC is a heterogeneous disease with diverse outcomes. Although many men with low-grade localized PC will have an indolent disease course and often die of unrelated causes, men with high-grade or metastatic PC have a much more aggressive disease course, and metastatic disease remains a fatal diagnosis. Advancements in biomarkers, targeted therapies, and combination systemic treatments aim to improve outcomes in men with PC by using a precision oncology approach to optimally tailor treatment to the unique biology of each patient.

PC is an androgen-dependent malignancy, and most first-line therapies target androgen production and the androgen receptor (AR) signaling axis. Androgens are secreted factors that develop and maintain male sexual reproductive tissues, including the prostate. The two most common androgens are testosterone and its downstream metabolite dihydrotestosterone (DHT) [2]. Androgens are primarily secreted by Leydig cells in the testis and a small amount is produced by the adrenal glands. AR is part of the steroid hormone receptor super family and is a nuclear transcription factor. Full length AR (AR-FL) contains a ligand-binding domain (LBD), a DNA-binding domain (DBD), a hinge region containing a nuclear localization signal, and a poorly conserved N-terminal domain (NTD) [3]. When AR-FL binds to DHT, it dimerizes and translocates to the nucleus where it binds to androgen receptor elements (AREs) along with coregulators to regulate AR-target gene expression [3]. AR supports prostate function by positively regulating the prostate-specific antigen (PSA), which is dysregulated in many PCs, and increased PSA serum levels are a common clinical indicator of aberrations in prostate function [4]. In PC, the AR-signaling axis promotes growth and the epithelial-to-mesenchymal transition (EMT), a hallmark of metastatic aggressive cancer [5].

**Figure 1:**
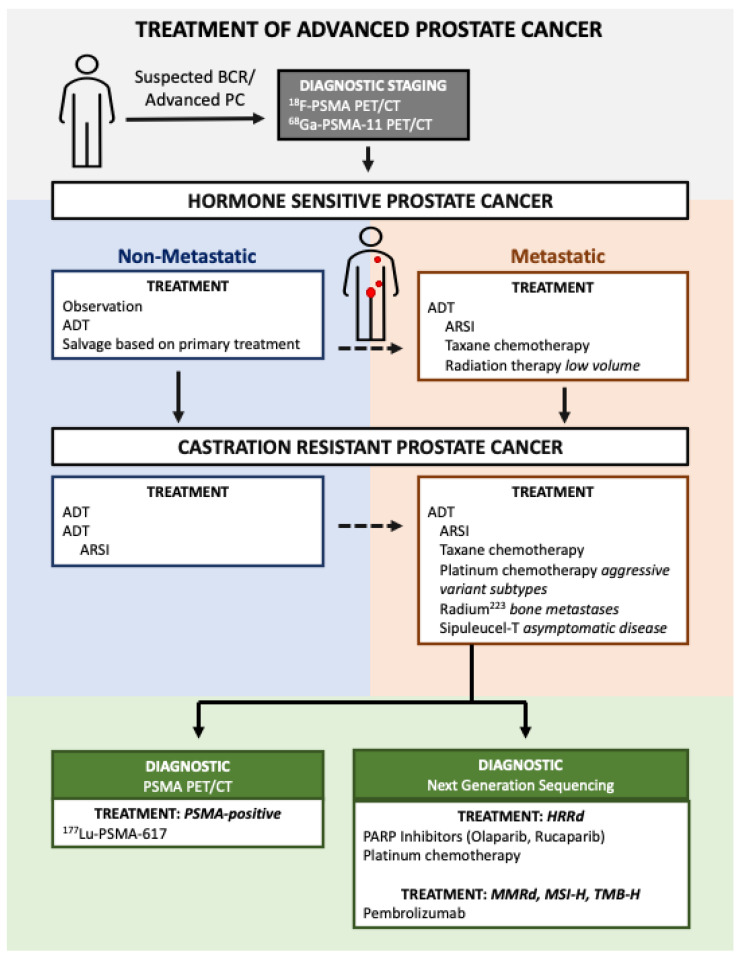
Treatment sequencing in recurrent/advanced prostate cancer. A core standard of care for metastatic disease is androgen deprivation therapy (ADT) in combination with at least one additional therapy. This review focuses on the therapeutic options upon disease progression following treatment. Abbreviations: biochemical recurrence, BCR; androgen deprivation therapy, ADT; androgen receptor-signaling inhibitor, ARSI; prostate-specific membrane antigen, PSMA; positron emission tomography/computational tomography, PET/CT; homologous recombination repair deficiency, HRRd; mismatch repair deficiency, MMRd; tumor mutational burden-high, TMB-H; microsatellite instability-high, MSI-H.

The first-line treatment for metastatic PC is androgen deprivation therapy (ADT) combined with an additional therapy such as an AR-signaling inhibitor (ARSI) and/or chemotherapy (Figure 1) [6]. Although treatment with ADT is highly effective in most patients, eventual resistance is almost universal—a disease state known as castration-resistant prostate cancer (CRPC). The past decade of research has seen an expansion of systemic therapies that target PC beyond the AR-signaling axis which have improved overall survival (OS) for patients with CRPC [7,8,9,10]. This review will cover advancements in PC biomarkers and systemic treatments, with a focus on novel targeted therapies. Specifically, we will focus on treatments for PC with homologous recombination deficiency (HRD), mismatch repair deficiency (MMRd), microsatellite instability (MSI), or elevated tumor mutational burden (TMB), and finally we will review advances in theranostics for the detection and treatment of PC with a focus on prostate-specific membrane antigen (PSMA)-directed therapy.

## 2. Advances in Molecularly Selected Therapies

Molecularly selected therapy is a treatment approach that utilizes a patient’s unique genetic or molecular signatures for the selection of optimal therapeutics, some of which can exploit well-defined molecular vulnerabilities within cancers. A significant advancement in precision oncology was the tissue-agnostic FDA approval of the programmed cell death-1 (PD-1) inhibitor pembrolizumab for the treatment of any tumor with mismatch repair deficiency (MMRd) or high microsatellite instability (MSI-H), regardless of the tissue of origin [11]. Since then, the indication for pembrolizumab has expanded to include any tumor with high tumor mutational burden (≥10 mutations/megabase; TMB-H). Two additional molecularly selected therapeutics—olaparib and rucaparib—were subsequently approved for the treatment of men with mCRPC and mutations in homologous recombination repair (HR R) genes. It is recommended that all men with mCRPC undergo somatic next-generation sequencing (NGS) to assess for MMRd, MSI, and HRD alterations [6]. Although the use of metastatic tumor tissue is the gold standard for NGS, the use of primary tissue is acceptable and advancements in cell-free DNA (cfDNA) technology have allowed blood-based sequencing for many patients [12,13,14]. cfDNA can accurately determine MMRd and MSI status, and detect actionable DNA repair gene alterations [15]. These approvals expanded the available treatments for men with mCRPC. In this section, we will review the evidence that led to these approvals and discuss their use in current practice.

### 2.1. Immunotherapy for Mismatch Repair Deficiency, Microsatellite Instability, and Elevated Tumor Mutational Burden

A repair mechanism crucial to genomic integrity is mismatch repair (MMR). Mismatched nucleotide incorporation is a consequence of damage (chemical/physical), imperfect DNA replication, and erroneous recombination between heteroallelic parental DNA [16,17]. MMR is a process that requires many genes to be executed properly, including *MSH2-6*, of which *MSH2* and *MSH6* are the most frequently altered MMR genes in PC [17]. MMR deficiency (MMRd) is associated with high tumor mutational burden (TMB-H) and high microsatellite instability (MSI-H) [18,19]. An estimated 3–5% of men with PC have MMRd, most likely derived from a somatic mutational event rather than a germline pathogenic alteration [20,21,22].

A consequence of MSI-H and TMB-H is the expression of strongly immunogenic mutant proteins called neoantigens, which are hypothesized to increase the efficacy of existing immunotherapies [23,24]. The generation of neoantigens by MMRd may increase susceptibility to immune checkpoint inhibitors (ICI) [25]. Immune checkpoints are key to inhibiting autoimmunity, or the targeted destruction of healthy cells. Tumors can co-opt immune checkpoints to prevent the immune system from targeting and destroying tumor cells, and ICI therapies can block these immune-suppressive interactions, allowing T cells to recognize and destroy cancer cells [25,26,27]. ICIs are indicated for use in diverse cancer types and include monoclonal antibodies that target the immune checkpoint proteins programmed death receptor-1 (PD-1), programmed death ligand-1 (PD-L1), and cytotoxic T-lymphocyte-associated protein 4 (CTLA-4) [28].

Despite being effective in many other cancers, men with unselected PC have had disappointing responses to checkpoint blockade therapies. The CTLA-4 inhibitor ipilimumab did not demonstrate an OS improvement in men with mCRPC in two randomized phase 3 trials [29,30]. Single-agent pembrolizumab, an anti-PD1 antibody, also resulted in low response rates (3–5%) in unselected patients [31]. Combination therapy with combined CTLA-4 and PD-1 blockade resulted in modestly higher response rates but with significant added toxicity [32]. A proposed mechanism behind this resistance is that the PC microenvironment has decreased infiltration by immune cell subtypes [33].

Pembrolizumab is approved for any tumor with MMRd or MSI, including PC [11]. A phase 2 trial found that across 12 tumor types, 53% of patients given PD-1 blockade therapy experienced a durable response, and the subsequent next-generation sequencing (NGS) of 12,019 cancers found that >5% of adenocarcinomas derived from 11/12 tissues were MMR deficient [34]. Subsequently, the KEYNOTE-158 phase 2 trial showed that pembrolizumab monotherapy elicited a robust tumor response in 29% of TMB-H PC patients, of which 57% had an enduring response of >12 months [31]. The FDA approved pembrolizumab in 2020 for patients with TMB-H cancers that are metastatic or unresectable and poorly responsive to other treatment (Table 1) [35]. The number of patients with PC was limited in the prospective trials, leading to pembrolizumab’s tissue-agnostic approval. Therefore, it was initially unknown how effective pembrolizumab would be for men with PC whose tumors were MMRd, MSI-H, or TMB-H. Several retrospective studies have attempted to answer this question. A retrospective analysis of patients with metastatic PC who received pembrolizumab treatment showed that patients with MMRd had a response rate of 53%, which is much higher than the previously reported 3–5% response rate in the larger unselected patient population [36]. Further, a subset of responders showed a durable response at 12 months of continual treatment [36]. This is congruent with other retrospective analyses as well [17,21,37,38]. Pembrolizumab is approved for use in patients who have failed at least one other treatment previously; however, the optimal timing and sequencing of pembrolizumab with other therapies is yet to be elucidated [39,40]. Pembrolizumab immunotherapy is a recent addition to PC treatment schemes that offers benefits to a subset of patients who harbor molecular signatures of MMRd/MSI-H/TMB-H who have already failed conventional therapeutic regimes. This marks a significant advancement in the use of gene signatures as biomarkers for tissue-agnostic therapeutics in oncology.

### 2.2. Theraputics Indicated for Homologous Recombination Repair Deficiency

Another therapeutically exploitable molecular signature is homologous recombination repair (HRR) deficiency. HRR is a high-fidelity repair mechanism that uses the sister chromatid as a template to exactly repair DNA double-stranded breaks (DSB) [47]. When HRR is defective because of deleterious mutations in HRR genes, such as *BRCA2*, *ATM*, *CDK12*, and *CHEK2*, cells must rely on more error-prone DNA repair mechanisms, which leads to an increased rate of mutations and genetic alterations [48,49]. Patients with HRR deficiency (HRD) have a higher lifetime risk of developing certain cancers such as breast, ovarian, and prostate malignancies [50]. In a study evaluating the prevalence of HRR mutations in metastatic PC, the prevalence was found to be 11.8% in the germline and 23% in somatic sequencing [49].

The reliance of HRD cells on compensatory, error-prone repair mechanisms can be exploited therapeutically. Blocking these compensatory repair mechanisms can increase the rate of catastrophic genomic instabilities and drive cancer cell death. This also makes cancers with HRD more sensitive to DNA damage agents, such as platinum-based chemotherapies, as they are also able to achieve catastrophic cytotoxicity more readily [51,52].

#### 2.2.1. Poly (ADP-Ribose) Polymerase (PARP) Inhibitors

Poly (ADP-ribose) polymerase (PARP) inhibitors (PARPi) block compensatory repair pathways (e.g., base excision repair), which generates a synthetic lethal effect in HRD patients [53,54]. The efficacy of PARPi in BRCA-altered tumors is recognized in other tumor types such as breast and ovarian cancers [55]. Patients with PC who have progressed after a second-generation hormonal agent (e.g., abiraterone acetate or enzalutamide) with HRD are potential candidates for two FDA-approved PARP inhibitors, rucaparib and olaparib [41,42].

Rucaparib is indicated for patients with germline or somatic *BRCA1* or *BRCA2* mutations [41]. Data to support rucaparib’s approval in this setting originally came from the phase 2 TRITON2 trial [56]. TRITION2 enrolled men with mCRPC who had deleterious HRR alterations and had progressed on one or two prior second-generation hormonal agents as well as at least one taxane-based chemotherapy. Rucaparib demonstrated effectiveness in the subpopulation of men with *BRCA1* or *BRCA2* mutations, with a PSA response rate of 54.8% and a confirmed objective response rate of 43.5%. Rucaparib showed limited efficacy in patients with non-*BRCA* HRR alterations [57]. For example, out of 49 patients with a pathogenic *ATM* alteration, only 2 (4.1%) achieved a PSA response. Similarly, 1/15 (6.7%) of patients with *CDK12* alterations and 2/12 (16.7%) of patients with *CHEK2* alterations experienced a PSA response. The number of men with alterations in other genes was limited, but a response was observed in patients who had mutations in *PALB2, FANCA, BRIP1,* and *RAD51B* [56]. The low response rates in the non-*BRCA* HRR genes limited rucaparib’s indication to *BRCA1/2* mutations only. The confirmatory TRITON3 phase 3 trial randomized men with mCRPC who had progressed on an ARSI and who had a *BRCA1*, *BRCA2*, or *ATM* mutation to either rucaparib or the physician’s choice of therapy (docetaxel or an alternative ARSI). Rucaparib therapy led to significantly longer radiographic progression-free survival (rPFS) compared to control therapy (11.2 months versus 6.4 months) [58]. Similarly to the prior data, the majority of the benefits seemed to be experienced by men with *BRCA* mutations [58].

Olaparib has a broader approval for men with mCRPC and pathogenic mutations in *BRCA1*, *BRCA2*, and 11 additional HRR pathway genes with mutations [10]. The PROfound phase 3 clinical trial enrolled men with mCRPC who had progressed on a second-generation ARSI. Men were randomized to receive either olaparib or the physician’s choice of enzalutamide or abiraterone and divided into two cohorts. Cohort A included men who had at least one alteration in *BRCA1*, *BRCA2*, or *ATM*, whereas cohort B included men who had alterations in any of the 12 other prespecified genes. Significant improvement in rPFS was noted in cohort A (7.4 months compared to 4.6 months) alone and the overall population of cohort A + B (5.8 months compared to 3.5 months). Given the benefit seen in the overall population, olaparib was approved for use in men with all HRR alterations studied, with the exception of *PPP2R2A* which showed evidence of possible harm in prespecified subgroup analysis.

Despite this broad approval, there remain uncertainties about the effectiveness of olaparib in men with non-*BRCA1/*2 alterations. In subset analyses of PROfound, the benefit in the overall population seems to be largely experienced by men with *BRCA1/*2 alterations. In men with *ATM* and *CDK12* mutations, there was no difference in rPFS between the olaparib and control groups [59,60,61]. The control arm of the PROfound trial may also have been suboptimal, as the majority of patients had already received at least one of the “physician’s choice” control therapies (i.e., enzalutamide or abiraterone) and there is known cross-resistance between these agents. In addition, 18% of patients in the control arm had received both abiraterone and enzalutamide previously [61]. Ultimately, further research is needed to more precisely define which patients may benefit from treatment with PARP inhibitors.

Although it is estimated that 20% of men with metastatic PC have a germline or somatic alteration in HRR, PARPi therapy remains unavailable for most patients with PC who are phenotypically normal for HRR. The activity of single-agent PARPi is minimal in patients without HRD; however, there has been considerable interest in searching for combination therapies that will sensitize or increase the effectiveness of PARP inhibitors in the unselected patient population. Studies of PARPi in combination with second-generation ARSIs have yielded conflicting results. The PROpel phase 3 trial evaluated the efficacy of olaparib and abiraterone in patients with mCRPC who had not received prior chemotherapy [60]. Combination therapy improved image-based PFS compared to abiraterone alone regardless of HRR status [60]. However, MAGNITUDE, a similar phase 3 trial, did not find a rPFS benefit in men without HRR mutations for the combination of niraparib (PARPi) and abiraterone [62]. Similarly, a phase 2 trial of veliparib (PARPi) and abiraterone failed to show a PFS benefit compared to abiraterone alone in a group of unselected men with mCRPC [63].

There is likely a subset of men with functional alterations in HRR, but without canonical alterations, who may benefit from PARPi treatment. Identifying and/or expanding biomarkers for PARPi response is an area of active investigation, and there are many ongoing efforts to identify tumor features and gene signatures that accurately identify HRD and potential responsiveness to genotoxic therapies [64,65]. The use of gene signatures to identify candidate patients has been a precedent in both ovarian and breast cancer. The ARIEL2 phase 2 trial found that patients with ovarian cancer with a loss of heterozygosity benefit from PARPi treatment [66]. In breast cancer, a statistical model (HRDetect) was developed to distinguish mutational signatures that could better predict HRD beyond *BRCA1/2* deficiency [67]. Similar classification frameworks are being developed and tested on PC (e.g., NCT04951492) [64].

#### 2.2.2. Platinum-Based Chemotherapeutics

Platinum-based chemotherapy (cisplatin, and carboplatin) is often used in small-cell or aggressive-variant PC, and sometimes in patients who have failed standard-of-care treatments [68]. The use of platinum-based chemotherapies is limited in standard PC adenocarcinoma. The oral platinum agent satraplatin prolonged PFS (11.1 weeks compared to 9.7 weeks) and increased the time until pain progression but did not prolong overall survival in a phase 3, randomized, placebo-controlled clinical trial [69,70]. Patients with DNA damage repair mutations may benefit from platinum chemotherapy as it directly damages DNA. Increased platinum sensitivity in *BRCA*-altered tumors has been demonstrated in other tumor types including breast and ovarian cancers [71]. In addition, exceptional platinum responders have been reported in patients with PC that have a *BRCA2* alteration [51,52,72]. In a large retrospective study of men with mCRPC who had received platinum chemotherapy (either alone or in combination with another chemotherapy agent), men with DNA repair alterations had higher PSA level decreases and soft tissue responses compared to men without DNA repair alterations. Responses in patients with *BRCA2* alterations were particularly promising, with a decrease in PSA from the baseline of at least 50% in 63.9% of patients and a soft tissue response in 50% of patients [52].

### 2.3. Sipuleucel-T Cellular Immunotherapy

Sipuleucel-T is an FDA-approved vaccine for the treatment of asymptomatic or minimally symptomatic mCRPC [9,35,73]. This vaccine generates an immune response targeted against prostatic acid phosphatase (PAP), an antigen upregulated in PC [9]. Patient-extracted antigen-presenting cells (APCs) are incubated ex vivo with a recombinant antigen protein which contains PAP, and then re-introduced to the patient. The proposed mechanism is that these APCs stimulate an immune response against PAP and subsequently high-PAP-expressing PC cells [74]. In clinical trials, it extended patient survival by 4.1 months [73]. The high cost of sipuleucel-T is considered a major obstacle to the accessibility and utilization of this treatment [74].

## 3. Theranostics in Prostate Cancer

‘Theranostics’ refers to agents which target radioisotopes to cancer-specific structures, typically a protein or antigen, for imaging and therapeutic purposes. In the case of imaging, a PET-compatible positron-emitting radionuclide with a half-life in the range of minutes to hours is used to label the radiopharmaceutical, such as fluoride-18 (^18^F) [75,76]. For therapeutic theranostics, the radiopharmaceutical is conjugated to a beta- or alpha-emitting radionuclide with a much longer half-life, which allows targeted radiation dosing [77]. The first theranostic agent to be used specifically for PC was radium-223 (^223^Ra), which was approved by the FDA in 2013 for the treatment of patients with mCRPC with symptomatic bone metastases [78]. Recent years have seen a resurgence in the development of theranostic agents, culminating in the development of prostate-specific membrane antigen (PSMA)-targeted agents for both diagnostic and therapeutic use in PC. PSMA is a cell-surface protein expressed by prostate cells and expression of PSMA is often increased in PC and PC metastasis [79]. While PSMA is also expressed by neural, neuroendocrine, kidney and small bowel tissue, it is expressed 12 times more by prostate tissue and is a highly specific biomarker [80]. Though typically more highly expressed in PC, expression of PSMA can be lost in poorly differentiated cells [81,82]. Herein, we will review the current state of theranostics for their use in the diagnosis and treatment of PC.

### 3.1. Diagnostics

Positron emission tomography (PET) scans have been applied in oncology for decades to search for tissues with an abnormal uptake of radiotracers [76,83]. Combining PET with computed tomography (PET/CT) or magnetic resonance imagining (PET/MRI) can also provide anatomical information. This imaging modality can be used to define both primary tumor sites as well as identify regional spread and metastatic sites. Historically, PET has relied heavily on several generalized radiotracers that exploit the increased metabolic activity of tumors. A radiolabeled glucose derivative, (18)F-2-deoxy-2-fluoro-D: -glucose (FDG)(^18^F-FDG), is a common radiotracer used for many malignancies; however, it lacks sensitivity and specificity for PC [84].

Another radiotracer used for diagnostic PET/CT is ^11^C-choline, which has several advantages over ^18^F-FDG in the detection of PC, including rapid blood clearance and rapid uptake by prostatic tissue. The rapid prostatic tissue uptake allows imaging to occur before the radiotracer accumulates in the bladder for excretion via urine, allowing clearer imaging of the pelvic region [85]. While ^11^C-choline may be used to detect biochemically recurrent (BCR) PC, it also suffers from a lack of specificity and can detect non-prostate pathology, such as renal cell carcinoma [86].

The radiotracer ^18^F-fluciclovine is FDA-approved to detect clinically suspected BCR PC that eludes detection by other conventional imaging methods [87,88]. The ^18^F-fluciclovine synthetic amino acid analogue is imported by glutamate transporters [89,90,91]. The sensitivity of ^18^F-fluciclovine was 90.2% in the original prospective trial, but the specificity was only 40% [92]. Later refinement of the imaging methodology (increasing the time to imaging from 5 min to 15 min) brought specificity up to 84.8% in subsequent studies [93].

While the broad-spectrum radiotracers described above have advantages and disadvantages, new prostate-specific radiotracers targeting PSMA are quickly becoming the standard of care. ^18^F-PSMA and ^68^Ga-PMSA are the two PSMA targeting radiolabels that are approved by the FDA for use in PC detection. ^18^F-PSMA is primarily used for the detection and staging of BCR PC and has several clinical advantages over ^68^Ga-PMSA, including increased availability in-clinic, and a higher positron yield with lower positron energy, which improves the contrast and decreases the noise in the images generated [94,95]. ^68^Ga-PMSA may be superior at detecting BCR disease and is a companion diagnostic for ^177^Lu-PSMA-617 treatment (as detailed in the next section) [96,97]. PSMA PET/CT provides highly specific imaging data and is presently indicated for the diagnosis and staging of BCR disease.

### 3.2. Therapeutics

#### 3.2.1. Radium-223 Dichloride

Radium-223 was the first approved theranostic for use in PC and is indicated for mCRPC which has spread to the bone. Radium-223 is a calcium mimetic that accumulates in areas of bone with increased turnover, as is characteristic of bone metastases frequently observed in PC [78]. The diagnostic counterpart for radium-223 is either bone scintigraphy with ^99m^Tc-MDP or PET/CT with ^18^F-NaF [98]. The Alpharadin in Symptomatic Prostate Cancer Patients (ALSYMPCA) trial showed an increase in OS of approximately three months and improved quality of life [99]. Importantly, radium-223 also prolonged the time until the first symptomatic skeletal event, defined as the use of palliative radiation to treat skeletal symptoms, symptomatic pathologic fracture, spinal cord compression, or surgery to treat a tumor-related orthopedic condition. The main limitation to the use of radium-223 is hematologic toxicity, especially in a heavily pre-treated population.

#### 3.2.2. ^177^Lu-PSMA-617

Lutetium Lu 177 vipivotide tetraxetan (^177^Lu-PSMA-617, Lu-PSMA) targets beta radiation to PSMA-positive cells. Beta emitters release electrons which damage DNA and typically travel farther through tissue than alpha particles do [100,101]. The VISION phase3 clinical trial showed that ^177^Lu-PSMA-617 prolonged PFS (median: 8.7 vs. 3.4 months) and OS (15.3 vs. 11.3 months) in patients with PSMA-positive lesions compared to the investigator’s choice of therapy [8]. Based on the results of this trial, ^177^Lu-PSMA-617 treatment was FDA-approved in May of 2022 for patients with PSMA-avid mCRPC who have previously received a taxane chemotherapy and an ARSI. This represented a major advance for patients with mCRPC and limited other treatment options; however, there remain many unanswered questions about the optimal approach to the use of ^177^Lu-PSMA-617. One criticism of the VISION trial was the very high initial dropout rate (56%) of the control arm, which was addressed and improved to 16% later in the study [102]. The standard-of-care (SOC) treatment arm of the study excluded chemotherapy, immunotherapy, and radium-223 therapy because of a lack of research into the safety of combining these agents with ^177^Lu-PSMA-617. However, with emerging evidence supporting the efficacy of doublet and triplet therapies, further investigation as to the safety of ^177^Lu-PSMA-617 with these therapies is imperative [102].

In the Australian TheraP phase 2 trial, men with mCRPC were randomized to ^177^Lu-PSMA-617 vs. cabazitaxel. Importantly, TheraP screened participants with both PSMA PET/CT and FDG PET/CT and only included patients with PSMA-positive disease who had no sites of discordant FDG avidity. Compared to cabazitaxel, ^177^Lu-PSMA-617 had a higher PSA response rate (66% vs. 37%) whilst eliciting fewer grade 3 or 4 adverse events (33% vs. 53%) [103]. ^177^Lu-PSMA-617 treatment also resulted in significantly improved PSA response and radiographic PFS. There is significant interest in the prognostic value of dual PET/CT with radiolabeled PSMA and ^18^F-FDG, with an early study suggesting that FDG-positive/PSMA-negative lesions are a negative predictor of OS in mCRPC patients undergoing radioligand therapy [104].

Additional applications of ^177^Lu-PSMA-617 are under clinical investigation. The PSMAfore phase 3 trial is an on-going investigation of the effect of ^177^Lu-PSMA-617 treatment compared to a change in ARSI therapy in taxane-naive progressive mCRPC patients, and has shown a significant PFS benefit compared to the control arm thus far [105,106]. The phase 3 trial, PSMAddition, is investigating ^177^Lu-PSMA-617 with the SOC compared to the SOC alone in patients with treatment-naive metastatic hormone-sensitive PC (HSPC) [107,108].

#### 3.2.3. ^177^Lu-PSMA I&T

There are several clinical trials in progress with another PSMA radioligand agent, ^177^Lu-PSMA I&T. The SPLASH and ECLIPSE phase 3 trials are evaluating ^177^Lu-PSMA I&T in patients with mCRPC who have previously failed ADT/ARSI [101]. However, there are concerns regarding increased renal radiation compared to that observed with ^177^Lu-PSMA-617 [109]. The BULLSEYE phase 2 trial was initially designed to evaluate ^177^Lu-PSMA I&T; however, an update to the clinical trial in 2022 changed the agent to ^177^Lu-PSMA-617. BULLSEYE is evaluating the use of ^177^Lu-PSMA-617 as an early therapeutic option in ARSI/chemotherapy-naive patients with metastatic HSPC [110].

#### 3.2.4. ^225^Actinium-PSMA-617

Very similar in concept is ^225^Actinium-PSMA-617 (Ac-PSMA), which emits low-dose alpha radiation. Alpha emitters are highly cytotoxic and mainly generate DNA double-stranded breaks. A small pilot study (*n* = 17) establishing a treatment protocol for ^225^Ac-PSMA-617 in chemotherapy-naive metastatic PC, yielded a >90% decrease in PSA levels in 82% of patients, and 41% of patients achieved remission for 12 months [111]. The AcTION (NCT04597411) phase 1 clinical trial is currently enrolling, and aims to evaluate Ac-PSMA in three experimental arms: (1) naive for radioligand therapy, prior ARSI and/or chemotherapy, (2) naive for radioligand therapy, ARSI, and chemotherapy and, and (3) prior radioligand therapy with no selection for ARSI or chemotherapy [112]. There is some concern regarding the increased adverse events seen with the use of Ac-PSMA, as early studies suggest it may have increased hematological toxicity and cause severe xerostomia; however, larger trials are necessary to investigate these early observations [113]. Further research will also elucidate whether or not Ac-PSMA may be an option for patients who have progressed after ^177^Lu-PSMA-617 therapy. There are also early studies on combination ^225^Ac-PSMA and ^177^Lu-PSMA-617 therapy, which suggest that the combination may enhance responses to PSMA-targeted radiotherapy while limiting xerostomia [114].

## 4. Conclusions

The diagnosis and treatment of PC has advanced significantly; however, metastatic PC continues to defy curative treatment options and remains a terminal diagnosis. The increased understanding of molecular drivers of PC has led to improvements in biomarkers and targeted therapies, which has ushered in a new era of precision oncology treatment.

Recent work exploiting vulnerabilities derived from deficient DNA damage repair responses have provided several therapeutic agents for advanced PC. Although immunotherapy in PC has underperformed compared to some other cancer types, the tissue-agnostic indication of pembrolizumab still marks a significant step forward in precision oncology and a valuable therapeutic option for the treatment of patients with MMRd/MSI-H/TMB-H PC. Likewise, PARP inhibitors have been proven to be effective in patients with HRD but are less so in unselected patients.

Theranostics have gained considerable traction in the diagnostic setting and show significant promise as therapeutic agents. PSMA-targeted radiotracers have high specificity and while they are primarily indicated for the detection of BCR disease, they are also showing promise in early diagnosis and staging. Coupled with alpha- and beta-emitting radioligand PSMA-targeted therapies, theranostics offer a highly specific radiation therapy option capable of treating PSMA-positive PC.

## 5. Future Directions

A fundamental challenge in the treatment of PC is the progression to castration-resistance. For men with mCRPC, there is an urgent need to meet this challenge and develop new therapeutic options. Targeting PC beyond the AR-signaling axis is clinically beneficial and the development of novel therapeutics or cross-application of current therapeutics used in other cancers can expand existing PC treatment options.

The advent of targeted next-generation sequencing panels allowed the identification of men with certain genetic biomarkers who would benefit from therapies such as checkpoint blockade, PARP inhibitors, or platinum chemotherapy. There is still much work to be carried out to ultimately achieve the goal of having precision medicine for every patient. Although therapies such as pembrolizumab and PARP inhibitors elicit robust responses in some patients, significant research efforts are needed to both further understand how to identify patients most likely to benefit from them and also to develop combination therapeutics to increase efficacy in unselected patients. Theranostics show immense promise as both diagnostic and therapeutic agents; however, many questions remain regarding the optimal timing and sequencing of these agents and the best way to utilize more sensitive diagnostic scans. In addition, future research is needed to identify predictive biomarkers for PSMA-targeting radioligand therapy.

The identification of biomarkers for molecularly selected therapeutics remains an area of significant interest. Additionally, many questions remain regarding the synergy and safety of newly approved therapeutics in doublet and triplet combination therapies. As these questions are answered, the field will grow closer to achieving the goal of optimally tailoring treatment to the unique biology of each patient.

## Figures and Tables

**Table 1:** FDA approval and indication of therapeutic and diagnostic agents.

Agent	Year FDA Approval	Supporting Clinical Trial	Indication
Pembrolizumab	2017 [11]	NCT02628067 (KEYNOTE-158)	Tissue-agnostic approval for unresectable or metastatic microsatellite-high (MSI-H) or mismatch repair deficiency (MMRd) solid tumors following prior treatment with no satisfactory alternative treatment available
	2020 [35]	NCT02628067 (KEYNOTE-158)	Tissue-agnostic approval for unresectable or metastatic cancer with tumor mutational burden-high (TMB-H)
Rucaparib	2020 [41]	NCT02952534 (TRITION2)	Patients with mCRPC with deleterious BRCA1/2 mutation who have progressed following androgen receptor-directed therapy and taxane chemotherapy
Olaparib	2020 [42]	NCT02987543 (PROfound)	Patients with mCRPC with deleterious or suspected deleterious BRCA1, BRCA2, ATM, BARD1, BRIP1, CDK12, CHEK1, CHEK2, fANCL, PALB2, RAD51B, RAD51C, RAD51D, and RAD54L mutations who have progressed following treatment with enzalutamide or abiraterone
^18^F-fluciclovine PET/CT	2016 [43]	NCT02578940 (FALCON)	Patients with suspected biochemically recurrent prostate cancer
^18^F-DCFPyL-PSMA PET/CT	2021 [44]	NCT02981368 (OSPREY)	Patients with suspected prostate cancer metastasis who are potentially curable via surgery or other therapy
	2021 [44]	NCT03739684 (CONDOR)	Suspected biochemical recurrence indicated by prostate-specific antigen levels
^68^Ga-PSMA-11 PET/CT	2020 [45]	NCT03368547	Patients with suspected prostate cancer metastasis who are potentially curable with surgery or radiation
	2020 [45]	NCT02940262	Suspected biochemical recurrence indicated by prostate-specific antigen levels
Radium^223^	2013	NCT00699751 (ALSYMPCA)	Patients with metastatic prostate cancer with symptomatic bone metastasis
^177^Lu-PSMA-617	2022 [46]	NCT03511664 (VISION)	Patients with PSMA-positive metastatic castration-resistant prostate cancer who have progressed following androgen receptor pathway inhibition and taxane chemotherapy

Abbreviations: metastatic-castration resistant prostate cancer, mCRPC; prostate-specific membrane antigen, PSMA.
